# Membrane Progesterone Receptor Beta Regulates the Decidualization of Endometrial Stromal Cells in Women with Endometriosis

**DOI:** 10.3390/ijms26157297

**Published:** 2025-07-28

**Authors:** Dora Maria Velázquez-Hernández, Edgar Ricardo Vázquez-Martínez, Oliver Cruz-Orozco, José Roberto Silvestri-Tomassoni, Brenda Sánchez-Ramírez, Andrea Olguín-Ortega, Luis F. Escobar-Ponce, Mauricio Rodríguez-Dorantes, Ignacio Camacho-Arroyo

**Affiliations:** 1 Unidad de Investigación en Reproducción Humana, Instituto Nacional de Perinatología-Facultad de Química, Universidad Nacional Autónoma de México, Ciudad de México 11000, Mexico; doravelazquezher@gmail.com (D.M.V.-H.); vamer@comunidad.unam.mx (E.R.V.-M.); 2 Departamento de Ginecología Quirúrgica, Instituto Nacional de Perinatología, Ciudad de México 11000, Mexico; oliverpco@gmail.com (O.C.-O.); drsilvestri@hotmail.com (J.R.S.-T.); dra.bsr@gmail.com (B.S.-R.); olguin.andrea@gmail.com (A.O.-O.); 3 Coordinación de Cirugía Pévica Avanzada, Instituto Nacional de Perinatología, Ciudad de México 11000, Mexico; drescobarponce@gmail.com; 4 Instituto Nacional de Medicina Genómica, Periférico Sur 4809, Ciudad de México 14610, Mexico; mrodriguez@inmegen.gob.mx

**Keywords:** progesterone, membrane progesterone receptors, decidualization, endometriosis, endometrial stromal cells

## Abstract

Endometriosis is a disorder characterized by the presence of endometrial tissue outside the uterus, leading to dyspareunia, chronic pelvic pain, dysuria, and infertility. The latter has been related to implantation failure associated with alterations in decidualization, a process regulated by sex hormones such as progesterone. Membrane progesterone receptor β (mPRβ) exhibits a lower expression in endometriotic tissues than in normal endometrial ones. However, the role of mPRβ in decidualization is unknown. This work aimed to investigate whether mPRβ plays a role in the decidualization of endometrial stromal cells (ESCs) derived from women with and without endometriosis. The mPR agonist OrgOD-2 induced the gene expression of key decidualization markers (insulin-like growth factor binding protein 1, prolactin, transcription factor heart and neural crest derivatives-expressed transcript 2, and fork-head transcription factor) in healthy ESCs, eutopic (uterine cavity), and ectopic (outside of the uterine cavity) ESCs from women with endometriosis. Notably, the expression of the decidualization markers was lower in endometriotic cells than in healthy endometrial ones. An siRNA mediated knockdown of mPRβ reduced the expression of decidualization-associated genes in ESCs treated with a decidualization stimuli, regardless of whether cells were derived from healthy women or those with endometriosis. Our data suggest that progesterone, through mPRβ activation, regulates the decidualization process in endometrial stromal cells from women with and without endometriosis.

## 1. Introduction

Endometriosis is one of the most common benign gynecological disorders affecting women of reproductive age [1]. It presents stromal and epithelial endometrial cells outside the uterine cavity, and the main symptoms of this disease include dyspareunia, chronic pelvic pain, dysuria, painful defecation, bloating, and constipation [1,2]. Moreover, approximately 25 to 40% of women with endometriosis present infertility, often linked to ovarian insufficiency, pelvic adhesions, and implantation difficulties [3]. RNA sequencing analysis has revealed that cells derived from eutopic (lining of the uterine cavity) and ectopic (outside the uterine cavity) endometrium of women with endometriosis exhibit a transcriptional profile associated with infertility [4].

Elevated estradiol levels characterize endometriosis, driving the growth and persistence of endometriotic tissue and inducing inflammation and pain [5]. Additionally, progesterone (P4) resistance in endometriosis contributes to lesion growth, chronic inflammation, aberrant gene expression, and a non-receptive endometrium [6].

Successful establishment of pregnancy requires endometrial decidualization, a process involving the morphological and functional transformation of endometrial stromal cells (ESCs) into decidua-like epithelial cells. Decidual cells express a wide range of molecules, including Heart and Neural Crest Derivatives-Expressed Transcript 2 (HAND2), Fork head Box O1 (FOXO1), Bone Morphogenetic Protein 2 (BMP2), a member of the Transforming Growth Factor-Beta (TGF-β) superfamily, Homeobox A10 (HOXA10), Insulin-Like Growth Factor-Binding Protein 1 (IGFBP1), Prolactin (PRL), and Zinc Finger and BTB Domain-Containing 16 (ZBTB16). These molecules enable immune cell recruitment, vascular remodeling, and stimulation of the endometrial glandular system to facilitate embryo implantation [7,8,9,10,11,12,13,14,15]. Defects in decidualization and embryo implantation have been associated with recurrent pregnancy loss, preeclampsia, and infertility [3,16]. Decidualization is a hormone-dependent process, with P4 as a key regulator, acting through its receptors, which are categorized into intracellular (classical) and membrane (non-classical) receptors [17,18]. Chromatin immunoprecipitation sequencing studies and gene expression profiling have demonstrated the role of intracellular P4 receptors (PR-A and PR-B) in regulating the expression of distinct sets of genes during decidualization and pregnancy establishment [19]. Furthermore, PR-A and PR-B regulate the expression of various transcription factors, such as AP-1, FOSL2, and JUN, which are pivotal in gene expression regulation during decidualization [20].

Previous studies have suggested that P4 effects on decidualization-associated gene expression are not solely attributed to PR activation in human immortalized ESCs, indicating the involvement of other regulatory mechanisms [21]. In the case of non-classical P4 receptors, decreased expression of the P4 receptor membrane component (PGRMC1) in human ESCs has been linked to accelerated decidualization, whereas its overexpression inhibits decidualization [22,23].

Membrane P4 receptors (mPRs) belong to the progestin and adipoQ receptors PAQR family and present five subtypes in vertebrates: PAQR 7 (mPRα), PAQR 8 (mPRβ), PAQR 5 (mPRγ), PAQR 6 (mPRδ), and PAQR 9 (mPRε). mPRs can respond to P4 by activating G proteins or interacting with adapter proteins such as PGRMC and amyloid adapter protein containing pleckstrin homology domain, phosphotyrosine binding domain, and leucine zipper motif 1 (APPL), activating multiple signaling pathways [24,25]. mPRs exhibit varying expression patterns in the endometrium of rodents, bovines, rhesus monkeys, and humans across the reproductive cycle. In humans, mPRα overexpression occurs during the secretory phase of the menstrual cycle, while mPRγ and mPRε transcripts are elevated in the proliferative phase and reduced in the secretory one [26,27]. Notably, mPRβ expression appears stable throughout the menstrual cycle, although a reduced expression has been observed in pathologies such as recurrent spontaneous abortions [28]. In endometriosis, decreased mRNA and protein expression of mPRβ has been reported in eutopic and ectopic endometriotic tissue compared to endometrial tissue from women without the disease [29]. Nevertheless, the involvement of mPRβ in the decidualization of ESCs from women with and without endometriosis remains unknown.

## 2. Results

### 2.1. Demographical and Clinical Data

No differences in demographic data were observed between women with endometriosis and women without the disease, as shown in Table 1. Notably, 100% of women without the disease had full-term pregnancies, whereas more than 50% of women with endometriosis experienced abortions.

### 2.2. Decidualization of Human ESCs

**Figure 1:**
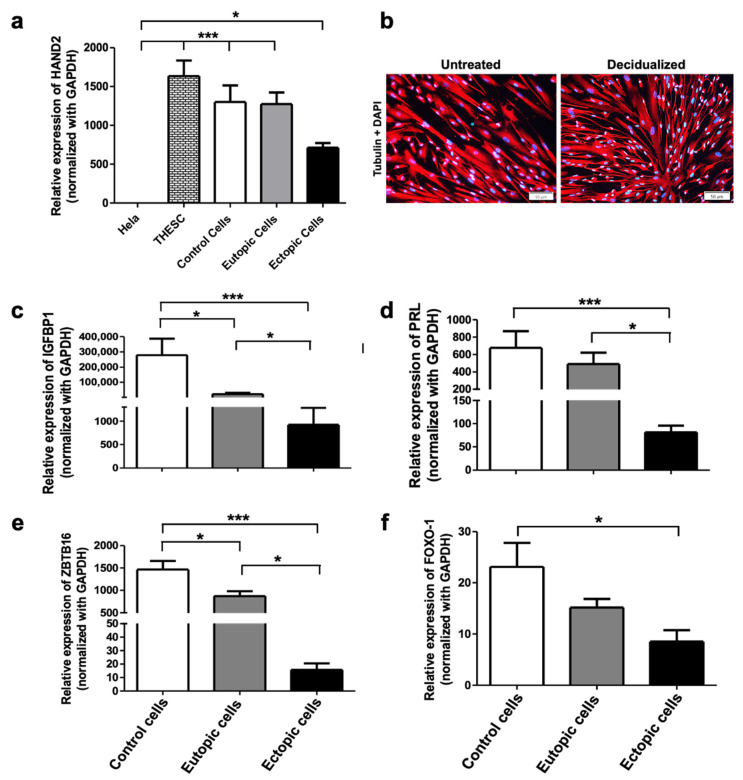
Decidualization of endometrial stromal cells from women with endometriosis. (**a**) Expression of *HAND2* in various cell types: Hela cells of epithelial origin, THESC cell line derived from the endometrium of healthy women, control cells (women without endometriosis), eutopic cells (obtained from endometriotic lesions in the uterine cavity), and ectopic cells (obtained from endometriotic lesions outside the uterine cavity). (**b**) Immunofluorescence: representative image of the decidualization of control cells treated with the decidualization cocktail (E2 + cAMP + P4) scale bar 50 μm. (**c**–**f**) Expression of decidualization markers *IGFBP1*, *PRL*, *ZBTB16*, and *FOXO1* in control, eutopic, and ectopic cells treated with the decidualization cocktail for 48 h. Data in the figures represent mean + SEM obtained from 10 independent experiments. (**a**) Statistical analysis was performed using one-way analysis of variance (ANOVA) followed by the Tukey post hoc test. (**c**–**f**) Statistical analysis was performed using Kruskal–Wallis followed by Dunns’s Multiple Comparison test. Statistical significance was considered as follows: * *p* ≤ 0.05 and *** *p* ≤ 0.001.

ESCs obtained from biopsies of women with or without endometriosis were isolated, cultured, and subjected to RT-qPCR assay to firstly analyze *HAND2* gene expression, which is highly expressed in endometrial stromal cells, to confirm that the primary cultures obtained from the biopsies were enriched in ESCs [8]. Additionally, stromal identity was further supported by immunofluorescence staining of vimentin, a mesenchymal marker, in representative cultures (Supplementary Figure S1). As depicted in Figure 1a, *HAND2* expression was detected in THESC cells (positive control), ESCs from women without endometriosis and in eutopic and ectopic ESCs from endometriosis patients. We observed a lower *HAND2* expression in ectopic cells than in the controls, consistent with findings previously reported [8]. Subsequently, stromal cells were treated with a decidualization cocktail (0.5 mM cAMP, 10 nM E2, and 1 µM P4). The transition from a fibroblast morphology to a rounded and cobblestone shape, characteristic of decidualized cells, is observed in Figure 1b. The expression levels of the decidualization markers *IGFBP1*, *PRL*, *ZBTB16*, and *FOXO-1* were assessed by RT-qPCR (Figure 1c–f). Notably, a reduced expression of IGFBP1 and ZBTB16 was observed in the eutopic cells, which was even more pronounced in the ectopic cells compared to control ones (Figure 1c–f).

### 2.3. mPRs Participation in the Decidualization of Human Endometrial Stromal Cells

**Figure 2:**
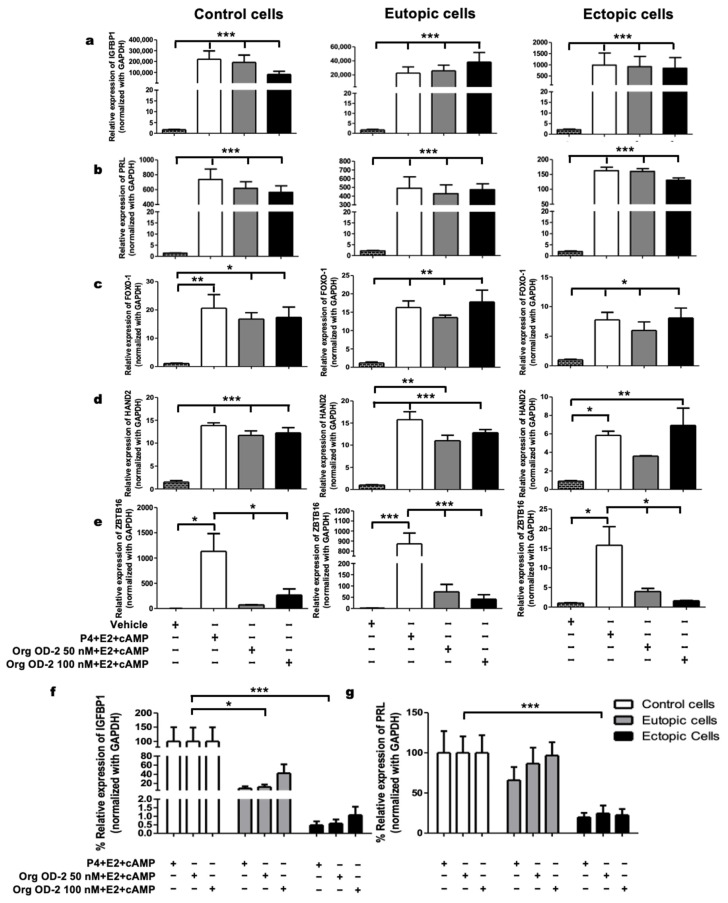
mPRs participate in the decidualization of endometrial stromal cells from women with and without endometriosis. (**a**) Expression of *IGFBP1 and* (**b**), *PRL* (**c**), *FOXO1*, (**d**) *HAND2*, and (**e**) *ZBTB16* in control, eutopic, and ectopic cells treated with vehicle, 50 nM and 100 nM of the selective mPR agonist Org OD2 + 10 nM E2 + 0.5 mM cAMP. The graphs (**f**,**g**) depict the comparison in percentage of expression levels of decidualization markers *IGFBP1* and *PRL* among control cells (women without endometriosis), eutopic, and ectopic cells derived from women with endometriosis. The data presented in the figures represent mean ± SEM obtained from 10 patients per group. Statistical analysis was conducted using the One-way ANOVA analysis followed by the Tukey post hoc test. Statistical significance was considered as follows: * *p* ≤ 0.05, ** *p* ≤ 0.01 and *** *p* ≤ 0.001.

To assess the role of mPRs in the decidualization of human endometrial stromal cells, we administered 50 nM and 100 nM of the selective mPR agonist Org OD 02-0 + E2 + cAMP. Both Org OD 02-0 concentrations mimicked the effects of P4 by upregulating the expression of *IGFBP1*, *PRL*, *HAND2*, and *ZBTB16* in control, eutopic, and ectopic cells from women with endometriosis (Figure 2a–d), suggesting the involvement of mPRs in the decidualization process of endometrial cells. Interestingly, in the case of the decidualization marker *ZBTB16*, none of the tested concentrations of Org OD 02-0 replicated the effect of P4 in control, eutopic or ectopic cells derived from women with or without endometriosis (Figure 2e). A 78% decrease in IGFBP1 expression in eutopic cells and a 99% decrease in ectopic cells compared to healthy endometrial stromal cells was observed (Figure 2f). Additionally, PRL expression was reduced by 77% in cells derived from ectopic tissue (Figure 2g).

### 2.4. mPRβ Induces the Decidualization of Human Endometrial Stromal Cells

**Figure A1:**
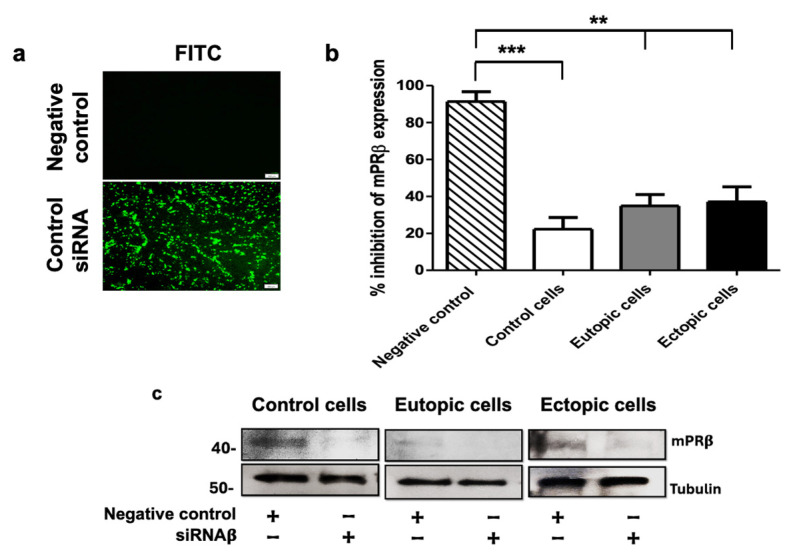
Transfection of control and mPRβ siRNA into human endometrial stromal cells. (**a**) Representative image of control cells transfected with control siRNA (FITC conjugate) for 7 h, scale bar: 100 μm. (**b**) Control, eutopic, and ectopic cells were transfected with mPRβ siRNA or negative control for 48 h. The percentage of transfection was evaluated by the expression of mPRβ using RT-qPCR. The data in the figures represent mean ± SEM obtained from 10 independent experiments. Statistical analysis was performed using a one-way analysis of variance (ANOVA) followed by the Tukey post hoc test. Statistical significance was considered as follows: ** *p* ≤ 0.01 and *** *p* ≤ 0.001. (**c**) Representative image of protein levels of mPRβ in control, eutopic, and ectopic transfected cells with mPRβ siRNA or negative control for 48 h.

To demonstrate the involvement of a specific mPR in the decidualization of endometrial stromal cells derived from control and women with endometriosis, we employed an mPRβ siRNA. We selected mPRβ due to its putative role in embryo implantation and its downregulation in the endometrium of patients with endometriosis [29]. We transfected control, eutopic, and ectopic endometrial stromal cells with mPRβ siRNA, negative control (scramble), or transfection efficiency control siRNA (FITC conjugate)-A. As depicted in Figure A1, siRNA transfection efficiency reached 90%, with mPRβ expression levels decreasing by 77.73% in control cells, 65.08% in eutopic cells, and 62.87% in ectopic cells compared to the negative control (Figure A1b). Protein levels were also evaluated, showing a decrease in cells transfected with siRNAβ compared to those transfected with the negative control (Figure A1c).

**Figure 3:**
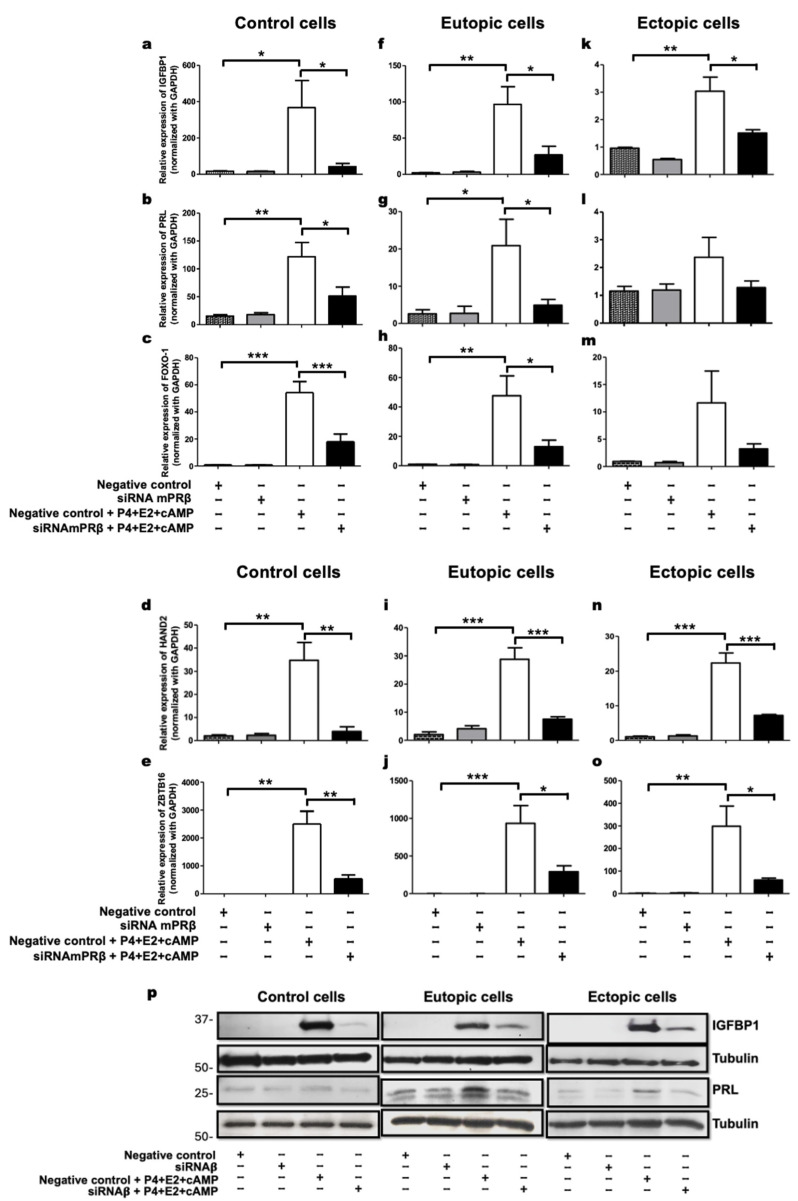
mPRβ induces the decidualization of human endometrial stromal cells. (**a**–**p**) Control, eutopic, and ectopic cells were starved for 24 h. Subsequently, they were transfected with mPRβ siRNA or negative control for 24 h, followed by treatment with the decidualization cocktail (10 nM E2 + 0.5 mM cAMP + 1 μM P4) for 24 h. The expression levels of the decidualization markers *IGFBP1*, *PRL*, *FOXO-1*, *HAND2*, and *ZBTB16* were evaluated by RT-qPCR and the protein levels of IGFBP1 and PRL were observed by Western blot. The data in the figures (**a**–**o**) represent mean and SEM obtained from 10 independent experiments. Statistical analysis was performed using one-way analysis of variance (ANOVA) followed by the Tukey post hoc test. Statistical significance was considered as follows: * *p* ≤ 0.05, ** *p* ≤ 0.01 and *** *p* ≤ 0.001 (**p**) Representative image of protein levels of IGFBP1 and PRL in control, eutopic, and ectopic cells transfected with mPRβ siRNA or a negative control for 24 h. Following transfection, cells were treated with the decidualization cocktail (0.5 mM cAMP + 10 nM E2 + 1 µM P4) for an additional 24 h.

Our findings demonstrate that the transfection with mPRβ siRNA resulted in a 60–80% reduction in the expression of the decidualization markers *IGFBP1*, *PRL*, *HAND2*, *FOXO1*, and *ZBTB16* in control cells (Figure 3a–e) and a 60–70% reduction in eutopic cells (Figure 3f–j) compared to the negative control + E2 + P4 + cAMP. In ectopic cells, a 50–80% decrease was observed in the expression of *IGFBP1*, *HAND2*, and *ZBTB16* compared to the same negative control (Figure 3k,n,o). For *PRL* and *FOXO1*, a decreasing trend in expression levels was noted in ectopic cells (Figure 3l,m). Additionally, the protein levels of IGFBP1 and PRL were reduced following transfection with mPRβ siRNA, confirming that mPRβ is essential for the decidualization process in cells from healthy women and women with endometriosis (Figure 3p).

## 3. Discussion

The human endometrium undergoes complex and dynamic changes to establish a suitable microenvironment for pregnancy. The decidualization of ESCs plays a crucial role in initiating and facilitating implantation [7]. While the involvement of PR in endometrial receptivity is well-established, recent interest has shifted towards non-classical receptors such as PGRMC1 and 2 and mPRs. Currently, limited information exists regarding the role of each receptor subtype in uterine function, prompting our investigation into whether mPRs contribute to the decidualization process of ESCs in women with and without endometriosis. Notably, P4 regulates the expression of decidualization markers such as *IGFBP1*, *PRL*, *FOXO1*, *HAND2*, and *ZBTB16* [7,8,9,10,11,12,13,14,30].

Our results demonstrate a decrease in the expression of decidualization markers in cells derived from women with endometriosis compared to those without the disease when exposed to P4 + E2 + cAMP, consistent with previous findings [31,32,33]. It has been proposed that P4 resistance in endometriosis may result from a decreased PR-B/PR-A ratio [34]. Interestingly, when a specific mPR agonist (Org OD 02-0) was added to the decidualization cocktail, a similar decrease in decidualization markers was observed (Figure 2f,g), suggesting a potential contribution of mPRs to P4 resistance in endometriosis [29].

The Org OD 02-0 compound provided insights into the involvement of some mPRs (e.g., mPRα, mPRγ, mPRε, or mPRβ) expressed in the endometrium [26,27] in the decidualization of ESCs from women with and without endometriosis. Subsequently, we sought to identify the specific mPR subtype involved in this process. While mPRγ and mPRε are upregulated in the proliferative phase [26,27], their involvement in decidualization was deemed unlikely due to the timing of this process in the menstrual cycle. Conversely, mPRα’s association with PGRMC1, whose role in decidualization is known, made it less suitable for investigation [22,23]. Consequently, mPRβ was selected due to its consistent expression throughout the menstrual cycle, higher expression compared to mPRα in the endometrium, and observed downregulation in women with recurrent abortions and endometriosis [28,29].

Our findings suggest that mPRβ regulates the expression of *IGFBP1*, *PRL*, *FOXO1*, *HAND2*, and *ZBTB16* during decidualization, as evidenced by decreased gene expression levels following mPRβ inhibition in control and eutopic cells. While the expression of *PRL* and *FOXO1* showed a trend towards reduction in ectopic cells, the effect was less pronounced compared to other decidualization markers. This observation suggests that additional receptors contribute to gene regulation in these cells.

Interestingly, *ZBTB16* does not exhibit increased expression levels in response to the agonist Org OD 02-0, a preferential agonist of mPRα. However, blocking mPRβ expression results in a more than 70% reduction in *ZBTB16* expression, reinforcing the notion that mPRβ is essential for decidualization in cells from healthy women and those with endometriosis.

## 4. Materials and Methods

### 4.1. Tissue Collection

This study included 20 participants with endometriosis, specifically ovarian and deep infiltrating types, diagnosed through laparoscopy and histological analysis, and 20 control women without the disease from the Instituto Nacional de Perinatología, Mexico City. In both groups, women were between 18 and 45 years old, presented regular menstrual cycles, and had not undergone hormonal therapy in the three months before sample collection. All participants provided informed consent for their participation in this study. Most samples from women with endometriosis and those free of disease were collected during the proliferative phase of the menstrual cycle. Eutopic tissue biopsies were obtained using a Pipelle cannula (Laboratoire CCD, Paris, France), while ectopic tissue was obtained with surgical scissors during laparoscopic surgeries. This study received approval from the Research, Biosecurity, and Ethics Committees of the Instituto Nacional de Perinatología (reference number 2019-1-26).

### 4.2. Primary Cell Culture of ESCs

Primary endometrial stromal cells were isolated, as described in a previous study [21]. Briefly, the endometrial (eutopic) and endometriotic (ectopic) tissues were minced using scalpel blades and incubated in Hank’s Balanced Salt Solution (composition: NaCl 140 mM, KCl 5 mM, CaCl_2_ 1 mM, MgSO_4_ 0.4 mM, MgCl_2_ 0.5 mM, Na_2_HPO_4_ 0.3 mM, C_6_H_12_O_6_ 6 mM) with 10 mg/mL type I collagenase (Gibco, Grand Island, NY, USA) and 1 mg/mL DNase I (Boehringer Mannheim, Mannheim, Germany) with agitation at 37 °C for 2 h. The digested tissue was passed through a 40 µm nylon cell strainer (FALCON, Chicago, IL, USA), which allowed the separation of stromal cells from epithelial and glandular fragments based on size. Stromal cells that passed through the strainer were collected and cultured in DMEM/F-12 supplemented with 10% FBS, antibiotic-antimycotic (Gibco, Life Technologies, Grand Island, NY, USA), and gentamicin (Gibco, Life Technologies, USA) at 37 °C and 5% CO_2_. All experiments were performed using primary cultures at passages 2 and 3.

### 4.3. Cell Lines

The THESC cell line (endometrial stromal cell line immortalized by reversible human telomerase transcriptase; ATCC CRL-4003, Manassas, VA, USA passage 22) was cultured in DMEM/F12 without phenol red (Gibco, USA), supplemented with 1.5 g/L sodium bicarbonate, 1.0 mM sodium pyruvate (Gibco, USA), 0.5% insulin-transferrin-selenium (ITS; Gibco, USA), and penicillin/streptomycin (Gibco, USA), along with 10% fetal bovine serum (FBS; Biowest, Heathfield, UK). The HeLa cells (passage 10) were cultured in DMEM (Gibco, USA), supplemented with penicillin/streptomycin (Gibco, USA), and 10% fetal bovine serum (FBS; Biowest, UK). Both cell lines were maintained in a controlled environment at 37 °C and 5% CO_2_. The cell line THESC was used as a positive control of ESCs, and the epithelioid cervix carcinoma cell line HeLa was used as a negative control.

### 4.4. Decidualization Treatments

Human endometrial cells were cultured in 6-well plates within phenol red-free DMEM/F-12 supplemented with 10% charcoal-stripped FBS (SH30068.03, CYTIVA, Marlborough, MA USA) and antibiotic-antimycotic (Gibco, Life Technologies, NY). A decidualization cocktail: 0.5 mM of cAMP (B7880 Sigma-Aldrich, St. Louis, MO, USA) + 10 nM of estradiol (E2) (E2758, Sigma-Aldrich, St. Louis, MO, USA) + 1 μM of P4 (P8783, Sigma-Aldrich, St. Louis, MO, USA) [21], or 50 or 100 nM of the mPR agonist 10-Ethenyl-19-norprogesterone (Org OD 02-0) (Axon 2085, Med Chem, Reston, VA, USA) was added to the culture media for 48 h to induce decidualization. Cells were washed twice with cold PBS, and the RT-qPCR technique was performed as described below in Section 4.5 to firstly observe the enrichment of ESCs in the culture with *HAND2* expression, and then to evaluate the expression of decidualization markers *PRL*, *IGFBP1*, *FOXO-1*, and *ZBTB16*.

### 4.5. RNA Isolation and Reverse Transcription-Quantitative PCR (RT-qPCR)

Total RNA was isolated from human endometrial stromal cells using QIAzol reagent (Qiagen, Valencia, CA, USA) according to the manufacturer’s protocol. Briefly, tissue samples were homogenized in QIAzol, followed by the addition of chloroform to induce phase separation. After centrifugation, the aqueous phase containing RNA was carefully collected, precipitated with isopropanol, washed with ethanol, and finally resuspended in RNase-free water. RNA integrity and purity were evaluated by agarose gel electrophoresis and spectrophotometry, respectively. Complementary DNA (cDNA) was synthesized from 1 μg of total RNA in a 20 μL reaction volume containing M-MLV reverse transcriptase (Invitrogen, Waltham, MA, USA), 10 mM of each deoxynucleotide (dNTPs) (Invitrogen, USA), and the appropriate buffer, according to the manufacturer’s instructions. The synthesized cDNA was subsequently used for quantitative real-time PCR (RT-qPCR) to amplify gene fragments corresponding to decidualization markers: PRL, IGFBP1, FOXO1, and ZBTB16. Reactions were carried out using PowerUp SYBR Green PCR Master Mix (Applied Biosystems, Vilnius, Lithuania) and detected with the CFX96 Touch Real-Time PCR Detection System (Bio-Rad, Hercules, CA, USA). The cycling conditions comprised 40 cycles of 95 °C for 15 s and 60 °C for 1 min. Each sample was amplified in triplicate, and data were analyzed using the ΔΔCt method, with GAPDH as the internal control. Primer sequences are provided in Table 2.

### 4.6. Immunofluorescence

ESCs were cultured on coverslips to visualize the decidualization. Cells were washed with PBS, fixed with 4% paraformaldehyde for 15 min, permeabilized with 0.1% Triton X-100, and blocked with BSA 1% (Promega, Madison, WI, USA) (Research Organics, Cleveland, OH, USA) for 1 h. Cells were incubated with an anti-β-tubulin antibody (ab7291, Abcam, Cambridge, UK) or anti-vimentin (ab92597 EPR3776) in a blocking solution at 4 °C overnight. After three washes with PBS, cells were incubated in the dark at room temperature with Alexa Fluor 594-conjugated secondary antibody (ab150116) or Alexa Fluor 488-conjugated secondary antibody (ab150097) for 1 h. This was followed by incubation with DAPI for 6 min. After washing, coverslips were mounted using Aqua-Poly/Mount. Cell fluorescence was examined using an inverted microscope (Olympus IX73 Tokyo, Japan).

### 4.7. mPRβ siRNA Transfection

Human endometrial cells were cultured in 6-well plates within phenol red-free DMEM/F-12 containing 10% charcoal-stripped FBS (35-072-CV, CA) and antibiotic-antimycotic (Gibco, Life Technologies, NY). siRNA transfections were performed in serum-free Opti-MEM (Gibco, Life Technologies, NY) using 35 pmol of PAQR8 siRNA (ID85315 Origene Technologies, Rockville, MD, USA) and universal scrambled negative control siRNA or positive control siRNA (FITC Conjugated, sc-36869 Santa Cruz Biotechnology, Dallas, TX, USA) and mixed with lipofectamine when the cells reached 80% confluence. After 7 h, transfected cells with positive control siRNA were observed under a microscope (Olympus IX73 Tokyo, Japan). The decidualization cocktail of 0.5 mM cAMP + 10 nM E2 + 1 μM P4 was added to the culture media for 24 h to induce decidualization. Subsequently the RT-qPCR assays were performed, as described in Section 4.5 to evaluate the expression of decidualization markers *IGFBP1*, *PRL*, *HAND2*, *FOXO-1*, and *ZBTB16*.

### 4.8. Western Blot

Protein was isolated from cells using RIPA buffer (50 mM Tris-HCl, pH 8, 150 mM NaCl, 1% NP-40, 1 mM EDTA, 0.1% SDS, 0.5% sodium deoxycholate) supplemented with a protease inhibitor cocktail (cOmplete™, EDTA-free, 11873580001, Roche, Mannheim, Germany). Then, 60 μg of protein was separated on a 10% SDS-PAGE gel (Invitrogen, Life Technologies, Carlsbad, CA, USA) and electrophoretically transferred onto nitrocellulose membranes (Bio-Rad, cat. 1620097). Membranes were blocked with 1% BSA (Promega, Madison, WI, USA) and incubated overnight at 4 °C with the respective primary antibodies: mPRβ (sc-50109 (n15), Santa Cruz Biotechnology), IGFBP-1 (PA5-78020, Invitrogen), and PRL (PA5-95712, Invitrogen). Detection was performed using the Immobilon Forte kit (Millipore, Burlington, MA, USA) and horseradish peroxidase (HRP)-conjugated secondary antibodies, including rabbit anti-goat (sc-2768, Santa Cruz Biotechnology), anti-mouse HRP (ab205719, Abcam), and anti-rabbit HRP (ab6721, Abcam). β-Tubulin (ab7291, Abcam) was used as a control to ensure equal protein loading across samples.

### 4.9. Statistical Analysis

Data were analyzed using either One-way ANOVA followed by post hoc Tukey’s and Kruskal–Wallis’s test followed by Dunns, using PRISM software (version 8.0; GraphPad) to determine differences between experimental groups. Statistical significance was set at *p* < 0.05.

## 5. Conclusions

This study is pioneering in demonstrating that mPRs, particularly mPRβ, are involved in the decidualization of stromal cells in women with and without endometriosis. Notably, the induction of decidualization using the mPR agonist Org OD 02-0 was lower in endometriotic cells than in healthy ones. This type of research is essential to elucidate the role of mPRs in infertility associated with endometriosis and emphasize that the expression or activity of non-classical P4 receptors can answer many questions associated with P4 resistance and problems in embryo implantation.

## Figures and Tables

**Table 1:** Clinical characteristic of the women included in this study.

Clinical Characteristics	Control (n = 20)	Women with Endometriosis (n = 20)
Age years (mean ± SD)	38.4 (± 6.6)	33.22 (± 9.7)
Number of women with full-term pregnancies	20	4
Number of women with abortions	2	11
Number of women in the proliferative phase during biopsy collection	13	11
Number of women in the secretory phase during biopsy collection	7	9

n: Number of women.

**Table 2:** Primers used in the present study.

GENE	FORWARD 5′3′	REVERSE 5′3′
*HAND 2*	GAGGAAGAAGGAGCTGAACGAA	GTCCGGCCTTTGGTTTTCTTG
*IGFBP1*	TCCTTTGGGACGCCATCAGTAC	GATGTCTCCTGTGCCTTGGCTA
*PRL*	CATATTGCGATCCTGGAATGAGC	TCCTCAATCTCTACAGCTTTGGA
*FOXO-1*	AGGDTTAGTGAGCAGGTTACAC	TGGACTGCTTCTCTCAGTTCC
*ZBTB16*	TTGTCTGTGATCAGTGCGGT	GCCATGTCAGTGCCAGTATG
*PAQR8*	AGGACGCAGCAAACAGGACA	GCC AACACAGGCAGGAATAA
*GAPDH*	GACAGTCAGCCGCATCTTCT	GCCCAATACGAC CAAATCCGT

## Data Availability

The datasets generated and/or analyzed during the current study are available from the corresponding author upon reasonable request.

## Supplementary Materials

The supporting information can be downloaded at: https://www.mdpi.com/article/10.3390/ijms26157297/s1.

## Abbreviations

The following abbreviations are used in this manuscript:

mPRβ	Membrane Progesterone Receptor β
ESCs	Endometrial Stromal Cells
HAND2	Heart and Neural Crest Derivatives-Expressed Transcript 2
FOXO1	Fork head Box O1
BMP2	Bone Morphogenetic Protein 2 a member of the Transforming Growth Factor-Beta (TGF-β) superfamily
HOXA10	Homeobox A10
IGFBP1	Insulin-Like Growth Factor-Binding Protein 1
PRL	Prolactin
ZBTB16	Zinc Finger and BTB Domain-Containing 16
PAQR	Progestin and AdipoQ Receptors
Org OD 02-0	10-Ethenyl-19-norprogesterone
cAMP	Cyclic Adenosine Monophosphate
