# Supplementary material for: Membrane Progesterone Receptor Beta Regulates the Decidualization of Endometrial Stromal Cells in Women with Endometriosis
Source: Int J Mol Sci. 2025 Jul 28;26(15):7297. doi: 10.3390/ijms26157297 (PMC12347855; doi:10.3390/ijms26157297)
Supplement: Supplementary file 1 [file ijms-26-07297-s001.zip › ijms-3722879-supplementary.pdf]

Supplementary figure S1

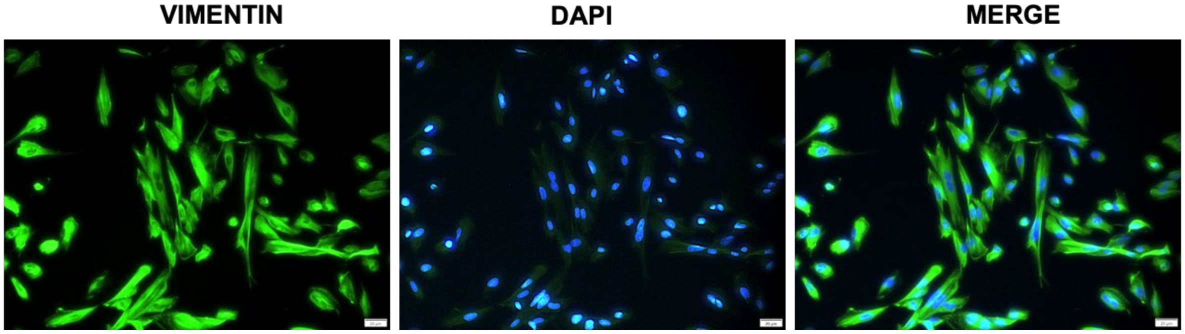

Representative image of cultured eutopic endometrial stromal cells stained with anti-vimentin antibody. Cells were fixed and incubated with a primary antibody against vimentin (green) and counterstained with DAPI to visualize nuclei (blue). Vimentin expression confirms the mesenchymal origin of the stromal cell population. Scale bar: 20 µm.
